# Prenatal Presentation of ENPP1‐Associated Generalized Arterial Calcification of Infancy at 15 + 1 Weeks: A Fetal Phenotype–Genotype Report

**DOI:** 10.1002/pd.70235

**Published:** 2026-08-12

**Authors:** Ismail Tekesin, Gunter Kerst, Loredana Delle Chiaie, April Dinwiddie, Heinz Gabriel

**Affiliations:** ^1^ Prenatal Unit Stuttgart Stuttgart Germany; ^2^ Center for Congenital Heart Defects Stuttgart Pediatric Intensive Care Pulmonology and Allergology Stuttgart Germany; ^3^ Clinic of Obstetrics and Gynecology Olgahospital Stuttgart Germany; ^4^ Center for Human Genetics Tuebingen Germany

**Keywords:** cardiovascular calcification, consanguinity, ENPP1, generalized arterial calcification of infancy

## Abstract

- What is already known about this topic?
  ◦ Generalized infantile arterial calcification type 1, caused by pathogenic variants in ENPP1, is a rare and severe genetic disorder characterized by extensive arterial calcification and high infant mortality.
  ◦ Most affected children are diagnosed postnatally.
- What does this study add?
  ◦ ENPP1‐associated GACI can present with widespread cardiovascular echogenicity as early as 15 + 1 weeks of gestation.
  ◦ Prolonged fetal stability does not exclude sudden late deterioration, and isolated cardiovascular echogenicity in a consanguineous pregnancy should prompt comprehensive genetic evaluation.

## 1 Fetal Phenotype

**FIGURE 1:**
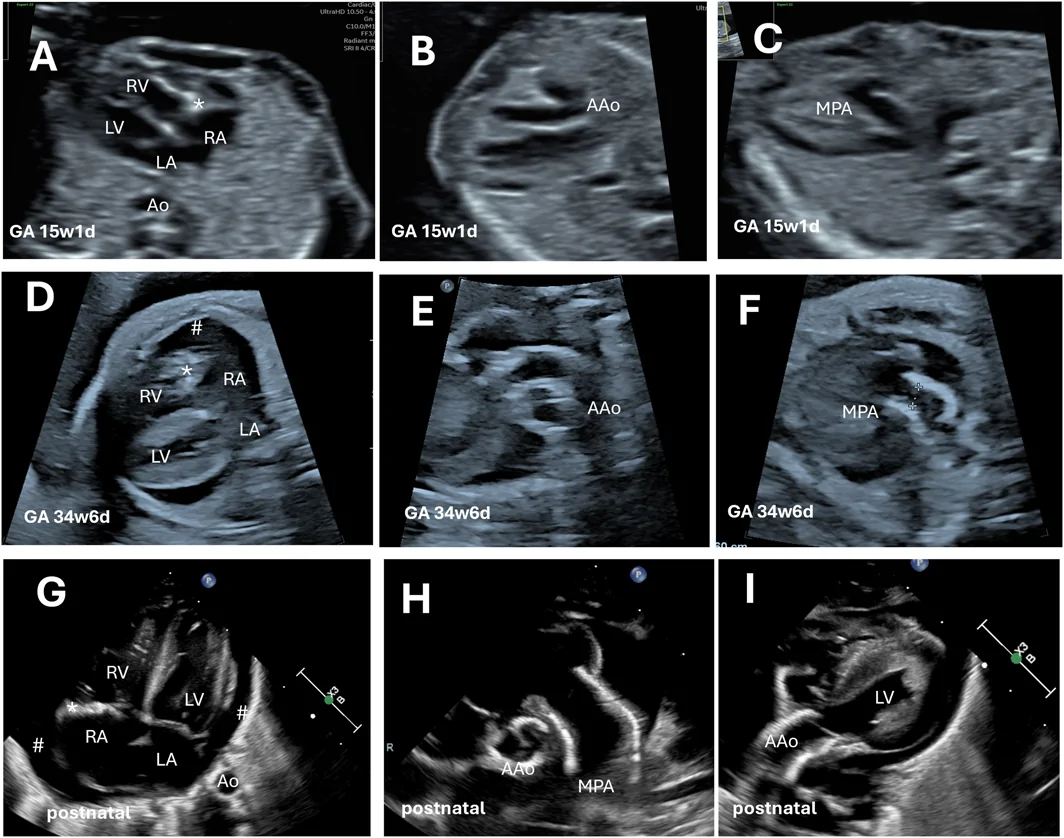
(A, D, G) Four‐chamber views, (B, E, H) left ventricular outflow tract views, and (C, F, I) right ventricular outflow tract views obtained prenatally at 15 + 1 weeks (A–C) and 34 + 6 weeks of gestation (D–F), and postnatally (G–I). Early fetal echocardiography demonstrated increased echogenicity of the tricuspid annulus (*), right ventricular endocardium, ascending aorta (AAo), and main pulmonary artery (MPA), while cardiac anatomy and ventricular function remained preserved. At 34 + 6 weeks, sudden clinical deterioration occurred with severe biventricular myocardial hypertrophy, markedly reduced ventricular contractility, and a large circumferential pericardial effusion (#). Postnatal echocardiography confirmed diffuse cardiovascular calcifications involving the coronary arteries, ascending aorta, and great vessels, associated with persistent myocardial hypertrophy and pericardial effusion. AAo, ascending aorta; Ao, aorta; LA, left atrium; LV, left ventricle; MPA, main pulmonary artery; RA, right atrium; RV, right ventricle.

A 31‐year‐old primigravida of Turkish origin in a consanguineous first‐degree cousin marriage was referred at 15 + 1 weeks of gestation after abnormal cardiac echogenicity was detected on routine ultrasound (Table 1A). Detailed fetal echocardiography revealed increased echogenicity of the tricuspid annulus, right ventricular endocardium, main pulmonary artery, and ascending aorta, with normal cardiac anatomy, ventricular size and function, and Doppler parameters. No pericardial effusion or extracardiac anomalies were identified (Figure 1A–C). Maternal infectious screening and autoimmune testing were negative, and non‐invasive prenatal testing excluded common aneuploidies. Trio‐exome sequencing was offered but declined. Follow‐up examinations at 20 + 5 and 30 + 5 weeks showed persistent cardiovascular echogenicity without structural or functional progression.

**TABLE 1A:** Clinical data.

Case	Parental details			Gestational age at diagnosis	Phenotypes (HPO terms)	Obstetric history	Outcome
1	Maternal Paternal	Age Ethnicity Age Ethnicity	34 Turkey 35 Turkey	15 + 1 weeks	Thickening of tricuspid annulus (HP:0031441) Myocardial hypertrophy (HP:0031319) Percardial effusion (HP:0001698) After birth: Vascular calcification (HP:0004934) Cardiovascular calcification (HP:0004934)	Primigravida Spontaneous pregnancy	Died at 6 weeks of age

At 34 + 6 weeks, rapid clinical deterioration occurred with severe biventricular myocardial hypertrophy, markedly reduced contractility, atrioventricular valve restriction, and a large circumferential pericardial effusion, as shown in Figure 1D–F, necessitating emergency caesarean delivery. The male newborn showed meconium‐stained amniotic fluid, low Apgar scores, and umbilical cord acidosis.

## 2 Diagnostic Method

Exome sequencing was performed on DNA from peripheral blood of the newborn using standard enrichment, sequencing, alignment, and variant‐calling pipelines. The mean sequencing coverage was 170×, and single‐nucleotide variants, small insertions/deletions, and copy‐number variants were assessed (Table 1B).

**TABLE 1B:** Clinical and genetic findings.

Procedure	Direct or culture	Test	Gene	Known disease (OMIM)	Variant	ACMG classification	Criteria applied	Zygosity	Interpretation
Post‐natal (EDTA blood)	Direct	Single exome sequencing	ENPP1, HGNC: 3356, transcript: NM_006208.3	Generalised infantile arterial calcification 1 (OMIM: 208000)	c.[2677G > T]; [2677G > T]; p.[Glu893*]; [Glu893*]	Pathogenic	PVS1 (strong) PS3 (supporting) PM2, PM3 PP4 (moderate)	Homozygous	Causative

## 3 Diagnostic Results and Interpretation

Conventional G‐banding karyotyping revealed a normal male karyotype (46,XY). Exome sequencing identified a homozygous ENPP1 c.2677G > T; p.(Glu893*) variant, which was classified as pathogenic (class 5) [1]. Both parents were confirmed to be heterozygous carriers of the variant, as summarized in Table 1B, and the phenotype was consistent with ENPP1‐associated generalized infantile arterial calcification type 1 (GACI1).

## 4 Pregnancy Outcome

Postnatal echocardiography confirmed diffuse calcifications involving the coronary arteries, great vessels, and cardiac valves, as shown in Figure 1G–I. Abdominal ultrasound demonstrated increased echogenicity of the abdominal aorta and its major branches. Despite intensive care management, the infant developed progressive cardiovascular failure and died at 6 weeks of age.

## 5 Discussion

Pathogenic variants of *ENPP1* are associated with a wide range of diseases. The *ENPP1* gene encodes ectonucleotide pyrophosphatase/phosphodiesterase 1, an enzyme that hydrolyses extracellular adenosine triphosphate (ATP) into adenosine monophosphate (AMP) and inorganic pyrophosphate (PPi). Pyrophosphate plays a key role in inhibiting pathological tissue calcification by preventing abnormal deposition of calcium and other minerals in the body [2]. Biallelic loss‐of‐function variants in *ENPP1* can cause generalized infantile arterial calcification type 1 (GACI1), less commonly autosomal recessive hypophosphataemic rickets type 2 (ARHR2), and pseudoxanthoma elasticum (PXE) [3]. Among these, GACI1 represents the most severe phenotype, characterized by early‐onset (in utero to infancy) calcification and/or stenosis of medium‐ and large‐sized arteries, leading to severe cardiovascular complications. Additional features may include periarticular calcifications, rickets, cervical spine rigidity, hearing loss, and skin manifestations resembling PXE. Prenatal findings may include polyhydramnios, hyperechogenicity associated with arterial and cardiac valve calcifications, hydrops fetalis, or pericardial effusion. Stillbirths and early neonatal deaths have been frequently reported [4, 5].

However, early ultrasound findings are often non‐specific and not immediately suggestive of an *ENPP1*‐related disorder such as GACI1. In the present case, a known pathogenic homozygous *ENPP1* variant was identified through trio exome sequencing in a neonate with severe biventricular myocardial hypertrophy, markedly reduced contractility, atrioventricular valve restriction, and a large circumferential pericardial effusion. The consanguineous parents were found to be heterozygous carriers. At 15 + 1 weeks of gestation, severe abnormal cardiac echogenicity was already detected on routine ultrasound; however, the parents declined further genetic testing at that time.

This case illustrates that GACI should be included among the differential diagnoses when cardiovascular calcifications or unexplained echogenicity of cardiac structures and great vessels are identified on fetal ultrasound, particularly in the setting of consanguinity. Trio exome sequencing has been shown to provide the highest diagnostic yield in fetuses with abnormal ultrasound findings [6]. Early genetic diagnosis enables more accurate prognostication and facilitates timely planning of neonatal management.

## Funding

The authors have nothing to report.

## Ethics Statement

Exome sequencing was performed as part of routine clinical care under standard clinical consent; this study involved retrospective analysis and data collection from patient records.

## Consent

Retrospective collection of data from patient records was exempted from additional informed consent as all clinical data contained in this report were anonymized.

## Conflicts of Interest

The authors declare no conflicts of interest.

## Data Availability

The data that support the findings of this study are available on request from the corresponding author. The data are not publicly available due to privacy or ethical restrictions.
